# Evidence of Incomplete Feeding Behaviors among South Carolina Tick Populations

**DOI:** 10.3390/insects15060385

**Published:** 2024-05-26

**Authors:** Kayla E. Bramlett, Laura E. Witt, Madeleine M. Meyer, Kia Zellars, Kyndall C. Dye-Braumuller, Melissa S. Nolan

**Affiliations:** 1 Department of Epidemiology and Biostatistics, Arnold School of Public Health, University of South Carolina, Columbia, SC 29208, USA; bramletk@email.sc.edu (K.E.B.); lewitt@email.sc.edu (L.E.W.); meyer7@mailbox.sc.edu (M.M.M.); zellars@mailbox.sc.edu (K.Z.); kyndallb@email.sc.edu (K.C.D.-B.); 2 Department of Environmental Health, Arnold School of Public Health, University of South Carolina, Columbia, SC 29208, USA; 3 Institute for Infectious Disease Translational Research, University of South Carolina, Columbia, SC 29208, USA

**Keywords:** incomplete feeding, blood meal, ticks, host, human, pathogen, vector-borne, tick-borne

## Abstract

**Simple Summary:**

In the southeastern United States of America, shifts in the environment such as climate change and host availability are pushing tick populations to spread into new areas. It is hypothesized that, as they migrate, tick populations have developed a behavior known as incomplete feeding. With this, ticks feed on more than one host at each life stage, increasing the chance of pathogen transmission. In South Carolina, we found evidence of ticks displaying this behavior. We collected engorged female ticks from stray dogs at animal shelters across the state in 2022. Testing showed that about a third of these ticks had fed on humans. The patterns varied depending on the tick species, where they were found, and the time of collection. This pilot study reflects the growing trend of tick-borne diseases in the southeastern USA. It is crucial to dig deeper into how factors like the season, location, and species are linked to incomplete feeding behavior in South Carolina’s tick populations.

**Abstract:**

Dynamic environmental conditions, such as climate change and host availability, have greatly influenced the expansion of medically relevant tick vectors into new regions throughout the southeastern United States of America. As tick populations migrate into new areas, it has been suggested they can exhibit a phenomenon known as incomplete feeding. With this phenomenon, tick vectors feed on more than one host at each life stage, thus increasing the likelihood of pathogen transmission. Although this behavior is not well understood, it presents an important threat to human health. Here we present evidence of incomplete feeding behaviors in multiple tick species in South Carolina. Engorged, blood-fed female ticks were collected from feral dogs at animal shelters across South Carolina in 2022. All ticks were tested for human blood meals using rapid stain identification blood tests. Approximately one third (33.78%) of all ticks tested positive for a human blood meal, with various patterns seen across species, geographic location, and collection month. The results of this pilot study follow the current national trend of increasing rates of tick-borne disease incidence in the southeastern United States of America and warrant further investigation into the relationship between seasonality, geographic distribution, species, and incomplete feeding among tick populations in South Carolina.

## 1. Introduction

The epidemiology of tick-borne diseases (TBDs) in the United States of America (USA) has dramatically changed over the last two decades. Human TBD incidence has doubled, and seven novel tick-transmitted pathogens have been identified nationally [1,2]. Moreover, according to the Centers for Disease Control and Prevention (CDC), upwards of five hundred thousand people are diagnosed and treated for some form of TBD every year, and this number is expected to rise in prospective years [3]. The increased incidence of federally reported TBDs can be attributed to the geographic range expansion of tick species into new regions [4]. Currently, there are nine tick species of medical and veterinary importance in the USA [5]. The most common among these have greatly expanded outside of their historic ranges, solidifying range expansion as a serious public health threat. For instance, *Amblyomma americanum*, one of the most abundant and common tick species in the USA, has gradually disseminated outside its historic range both north and westwards [6]. In the past two decades, *Ixodes scapularis* has significantly expanded into several Midwest, northeast, and mid-Atlantic states, while remaining an established species in the southeastern USA [7]. Additionally, *Amblyomma maculatum*, a tick species most found along the southern Atlantic coast, has steadily expanded its range into the mid-Atlantic and is projected to continue this expansion northwards [8].

Historically, tick vectors had well-defined geographic ranges due to local abiotic environmental factors and host–vector associations [6]. Therefore, the geographic expansion of ticks in the USA is believed to be heavily impacted by climate change, which transforms the natural environment, ultimately impacting these ecologic communities and relationships [3]. Different vector adaptation processes allow various tick species to establish themselves in these new domains [6]. One adaptation of importance is incomplete feeding, also referred to as interrupted or partial feeding. Ticks have four distinct life stages: egg, larva, nymph, and adult [9]. Once the eggs hatch, larvae must take a blood meal to molt to the nymphal stage; nymphs must take a blood meal to molt into an adult; and adults must take a blood meal to propagate [9]. Most ticks of public health importance in the USA, including *A. americanum*, *A. maculatum*, *I. scapularis*, *Ixodes keiransi*, *Dermacentor variabilis*, and *Rhipicephalus sanguineus*, are considered three-host ticks [9,10]. Three-host ticks hunt, attach, and feed on a different animal reservoir at every life stage [9,10]. With this behavior, incomplete feeding can occur if a tick detaches from its host before completion of a blood meal [11].

This behavior could be a sign of new ticks into a new area they have not previously inhabited. Both host type and atmospheric conditions are key factors that have been shown to influence how many hosts a tick will utilize throughout their life cycle [12]. It has been shown that adult female *I. ricinus*, *D. reticulatus*, and *Hyalomma asiaticum* ticks are able to detach from a host mid-blood meal and reattach to a different host, so long as they have not reached the critical mass needed to reproduce [12]. However, this phenomenon remains poorly studied, especially in ticks native to the USA. Therefore, the current study aims to explore the potential factors related to the increased number of tick populations across South Carolina (SC) by (1) testing the hypothesis that engorged female tick species are exhibiting incomplete feeding and (2) analyzing potential risk factors associated with these ticks. To address these aims, blood meal analysis was performed on host-attached ticks collected from feral dogs presenting at animal shelters throughout the state. 

## 2. Materials and Methods

### 2.1. Tick Collection and Processing

From January 2022 to July 2022, ticks were collected through an ongoing statewide tick surveillance project that included a partnership with animal shelters throughout SC. Participating animal shelters were located in all four regions of SC: the Upstate, located in the upper half of the state; the Midlands, located in the middle of the state; the Lowcountry, located on the lower coastline bordering Georgia (GA); and PeeDee, located on the upper coastline and bordering North Carolina (NC). At the start of each month, participating animal shelters were given a new ‘tick kit’ containing a mason jar filled with 75% ethanol, a data log, and a prepaid mailer. The shelters were instructed to place any ticks removed from a feral dog into the jar and fill out the data log upon doing so. The data log collected data on date, host species, and the number of ticks removed. Once the jar was received, the ticks were identified morphologically using published pictorial keys [13,14,15]. Each individual tick was then stored in a new 15 mL centrifuge tube and labeled with the collecting animal shelter, date, species, sex, life stage, and engorgement status. These ticks were stored at room temperature in 75% ethanol on the lab bench away from sunlight until further processing. Each individual tick was then bisected vertically using a scalpel sterilized in a Micro Bead Sterilizer at 300 °C. Half of each individual tick was placed in a new 15 mL centrifuge tube and stored in a −80° freezer to be used for pathogen testing. The other half was used for blood meal analysis.

Only engorged adult female ticks were included in this study. Although all life stages and both sexes blood-feed, only adult female ticks become fully engorged, increasing their body weight by almost 100 times their unfed weight [16,17]. Therefore, engorged adult female ticks exhibit the highest likelihood of completing a substantial blood meal.

### 2.2. Blood Meal Analysis

As the ticks used for this study were removed directly from a canine host, canine blood meal testing was not conducted. Instead, it was inferred all ticks were positive for a canine blood meal. However, rapid stain identification (RSID) blood test kits were used to test for the presence of human blood according to the manufacturer’s instructions (Independent Forensics, Lombard, IL, USA). Individual tick halves were scored across the inside of its body cavity using a disinfected and sterilized scalpel to remove a sample of coagulated blood. Each coagulated blood sample was then added to the buffer solution provided by the test kit in its own well. The samples were covered and left undistributed at room temperature for one hour to allow the coagulated blood to soften and dissolve. Once mostly dissolved, thin swabs were used to crush up and mix in any remaining congealed pieces. Next, 100 μL of each well mixture was transferred to the sample well of the test cassette using a sterile micropipette tip. The tests were allowed to sit at room temperature, undisturbed for 10 min before the results were read. The results were interpreted per the test kit’s instructions: visible red lines at both the control (C) and test (T) position indicated a positive test. All other results were considered negative.

### 2.3. Pathogen Testing

Tick DNA was extracted using a Qiagen DNEasy kit (Qiagen Sciences Inc., Germantown, MD, USA), and dsDNA was collected and purified using the QIAcube HT (Qiagen Sciences Inc., Germantown, MD, USA) as previously described by Dye-Braumuller, 2023 [18]. Finally, ticks were screened for various pathogens using a QuantStudio 5 Real-Time PCR System (Appendix A). *Amblyomma* and *Dermacentor* tick species were tested for spotted fever group *Rickettsioses* (SFGR, *R. parkeri*, *R. amblyommatis*, *R. rickettsii*, and SFGR general *ompA*) and *Ehrlichia* species (*E. ewingii*, *E. chaffeensis*, and Panola Mountain *Ehrlichia*), while *Ixodes* spp. and *Haemaphyalis longicornis* ticks were tested for *Borrelia burgdorferi*, *Anaplasma phagocytophilum* and *Theileria orientalis.*

### 2.4. Statistical Analysis

Descriptive statistics were performed to describe the ecological differences between human blood meal positives vs. negatives. Univariate logistic regression was performed to statistically assess the variance between feeding groups. Six multinomial logistic regressions were run for each category to accommodate for potential dilution effects of low counts within individual category variables. Variables with a *p*-value < 0.05 were considered statistically significant. Statistical analysis was conducted using both SAS OnDemand for Academics v3.81 (SAS Institute Inc., Cary, NC, USA), and Stata SE v15.1 (State Corporation, College Station, TX, USA). Finally, a geospatial map of human blood meal-positive ticks was created using ArcGIS Pro (Esri Corp, Redlands, CA, USA).

## 3. Results

A total of 225 eligible animal shelter ticks were received from 8 different veterinary practices across SC between January 2022 and July 2022. Only fully fed adult female ticks were included in the final analysis. Of these, 48% (n = 108) were Prostriata (*Ixodes* spp. only), and 52% (n = 117) were Metastriate (*Amblyomma*, *Dermacentor*, and *Haemaphyalis* spp.). Furthermore, six different tick species were obtained: 39.6% (n = 89) were *I. scapularis*, 26.7% (n = 60) were *D. variabilis*, 17.8% (n = 40) were *A. americanum*, 8.4% (n = 19) were *I. keiransi*, 5.3% (n = 12) were *H. longicornis* and 2.2% (n = 5) were *A. maculatum*.

Overall, 33.8% (n = 76) tested positive for a human blood meal (RSID positive). Of these, 34.2% (n = 26) were *D. variabilis*, 32.9% (n = 25) were *I. scapularis*, 14.5% (n = 11) were *A. americanum*, 10.5% (n = 8) were *I. keiransi*, and 7.9% (n = 6) were *H. longicornis*. No *A. maculatum* ticks were found to be positive for a mixed blood meal.

Few variables were found to be statistically associated with human blood meal-positive ticks in the univariate analysis (Table 1). Despite the diversity among the study population, genus and species were not likely risk factors associated with incomplete feeding. Ticks received in the month of March, however, had 2.26 times greater odds (*p*-value = 0.027; CI: 1.10 to 4.65) of testing positive for a mixed blood meal compared to those collected in all other months.

**Figure 1:**
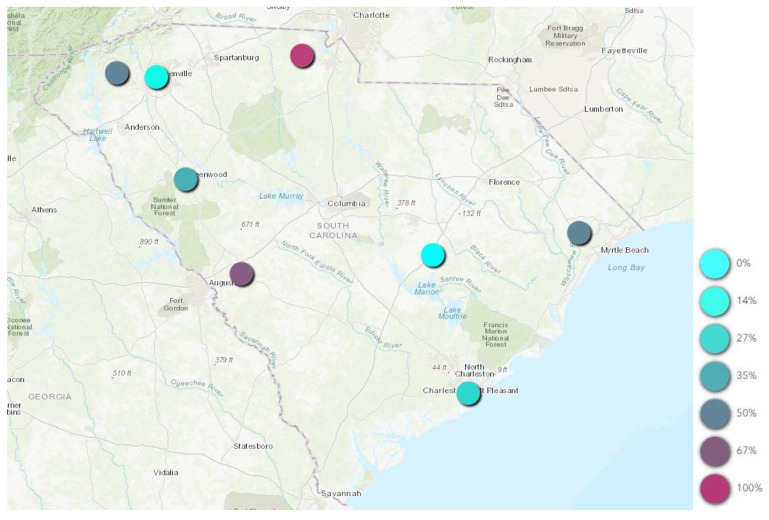
Geospatial distribution of percent mixed blood meal-positive adult ticks by county.

When assessing general location, 40.8% (n = 31) of positive ticks came from animal shelters in the Upstate, while only 2.6% (n = 2) came from animal shelters in PeeDee. However, no statistically significant association was observed between mixed blood meal-positive ticks and region. When evaluating location, county as a collective variable (*p*-value = 0.012), and Horry County (*p*-value = 0.027; OR: 2.26; CI: 1.10 to 4.65) were significant. In addition, Charleston (*p*-value = 0.068) and Greenville (*p*-value = 0.13) both neared significances. Taken together, this indicates that while region does not appear to be a risk factor, the county a tick was collected from potentially plays a big role in predicting if that tick was human blood meal-positive or not. Further, when looking at the rate of RSID positive ticks by county, 100% of ticks collected from animal shelters in York County, 67% from Aiken, 50% from Horry and 50% from Pickens were human blood meal-positive (Figure 1).

Overall, 48.7% (n = 37) of human blood meal-positive ticks, and 45.6% (n = 68) of human blood meal-negative ticks, tested positive for one or more pathogens. Although the infection rate for both mixed blood meal-positive ticks, and mixed blood meal-negative ticks were similar, pathogen testing was not statistically significant. This indicates being infected with a pathogen does not affect if a tick exhibits incomplete feeding or not.

## 4. Discussion

The current study aimed to (1) test the hypothesis that engorged female tick populations in SC are exhibiting incomplete feeding, and (2) analyze the risk factors associated with mixed blood meal-positive ticks. This investigation revealed calendar month and collection county may be important risk factors of incomplete feeding behavior. Moreover, the highest rate of human bloodmeal-positive ticks were from counties bordering both NC and GA, suggesting vector control differences between states may impact tick blood meal selection. Although tick genus and species were not significant, the results show *I. scapularis* and *D. variabilis* were most likely to demonstrate incomplete feeding. Moreover, while pathogen testing was not a significant risk factor associated with incomplete feeding in this pilot study, previous literature suggests pathogen-induced behavior changes may play a role in this. Taken together, the current study shows several preliminary results that can be built upon.

Although incomplete feeding among tick populations in SC is poorly studied, existing literature on other tick species and vectors can still give insight into potential risk factors that might influence this behavior. A study conducted in Switzerland found 19.5% of nymphs and 18.9% of adult *I. ricinus* ticks contained DNA from more than one vertebrate host [19]. Similar observations were recorded in St. Louis, Missouri where 16.2% of *A. americanum* nymphs tested positive for a mixed blood meal [20]. Another study in Pennsylvania suggested previously fed adult and nymphal *H. longicornis* ticks continued to quest for a new host despite their engorgement status [21]. In addition to ticks, this behavior has been widely described in other common vectors such as mosquitoes, sandflies, and triatomines. For instance, multiple studies have shown various mosquito species including: *Culex tritaeniorhynchus*, *Cx. quinquefasciatus*, *Anopheles coustani*, *An. pharonesis*, *An. funestus*, and *An. sacharovi*, feed on more than one host within the same gonotrophic cycle [22,23,24,25,26,27]. Furthermore, a study conducted on *Aedes aegypti* and *A. albopictus* mosquitoes found multiple blood meals were more likely to meet the insect’s nutritional needs [28]. A study conducted in the Republic of Tunisia discovered mixed blood meals in *Phlebotomus perniciosus* [29]. Similarly, mixed blood meals were detected in both *P. perniciosus* and *P. ariasi* sandflies in Spain [30]. A study from Maleki-Ravasan et al. found the presence of a mixed blood meal in field-captured sandflies [31]. In closing, incomplete feeding is not a phenomenon restrictive to our tick population, further validating the study’s findings.

A tick’s life cycle and feeding behavior are greatly influenced by their surrounding environment, particularly seasonality and ecology [6]. Although just under half of all ticks were collected in the month of May, only ticks collected in March were statistically significantly associated with a higher odds of a mixed blood meal, and thus incomplete feeding. Half (n = 9) of all dual blood meal-positive ticks collected in March were *I. scapularis*, while 27.8% (n = 5) were *I. keiransi*, and 22.2% (n = 4) were *D. variabilis*. In the southeastern USA, adult *I. scapularis* ticks commonly emerge in October but remain active throughout the winter, with females typically laying eggs in the spring [32]. Unlike *I. scapularis*, however, adult *I. keiransi* overwinter and begin questing in March [33]. Furthermore, *I. keiransi* females most commonly lay eggs in the late summer or early fall months [33]. Finally, adult *D. variabilis* ticks are most active between March and August, but typically peak in May and June [34,35]. Although there are minor differences in the seasonality of these species, all three are active in the month of March. Given these overlapping biotic factors combined with March representing a transitional climate month, the mix of species competition and abiotic uncertainly could be a driver for incomplete feeding behaviors.

Location was determined to be an important factor for mixed blood meal ticks. First, county as a collective variable was significant, suggesting location may be an important predictor for incomplete feeding in SC. Horry County was the only individual county with a statistically significant relationship with incomplete feeding. Most interestingly, the highest rate of mixed blood meal-positive ticks were collected from counties boarding surrounding states. For example, Horry, Pickens, and York counties, all which border NC, had mixed blood meal rates of 50%, 50%, and 100%, respectively. Similarly, Aiken, which borders GA, had a positive infection rate of 66.7%. Although every state has their own policies regarding vector control, SC is heavily underfunded. Surrounding states receive up to 2.5 times more financial assistance for vector and tick-borne disease-related programs [36]. As a result, tick surveillance and intervention programs are less common, making it easier for tick populations to expand and establish in new areas.

Previous studies have shown ticks originating from the north are genetically distinct from those originating from the south in the USA, although the current paper did not explore this [37,38]. This distinction is useful in not only pinpointing where tick populations originate from, but also if they have expanded outside their historic range. For example, Monzon et al. (2016) showed *A. americanum* ticks from New York and Oklahoma were distinct not just from each other, but also from historic range populations in North and South Carolina [37]. Similarly, a study from Xu et al. (2020) found northern (North Carolina, Virginia, Delaware, New Jersy, Rhode Island, Massachusetts, and New Hampshire) and southern (Florida, Georgia, and South Carolina) *I. scapularis* ticks had different 16S haplotype distributions [38]. The current study provides the groundwork for future, more in-depth, studies about these ticks’ distinct linages and migration patterns. Understanding these distinct lineages will allow for a more detailed comprehension of how geospatial and migratory patterns influence incomplete feeding.

*Ixodes scapularis* and *D. variabilis* were also the two most prevalent species to test positive for a mixed blood meal. Although *A. americanum* is regarded as the most abundant and aggressive human biting tick in the USA, two separate studies from Felz et al. (1996, 1999) demonstrated *D. variabilis* and *I. scapularis* are the second and third most common human-attached ticks [39,40]. In addition, several studies have directly demonstrated incomplete feeding among *I. scapularis* ticks in both controlled and natural conditions. Piesman et al. (1991) demonstrated that 38% of semi-engorged *I. scapularis* larvae, who previously detached from a dead host, reattached to a new host, and feed to completion [41]. A similar study observed semi-engorged *I. scapularis* nymphs voluntarily reattached to a new host and fed to repletion after spending up to 48 h on their first host [42]. However, fewer reports of incomplete feeding among *D. variabilis* ticks have been reported. Although one study from Reese et al. (2010) reported incomplete feeding in nymphal *D. variabilis* ticks, the authors did not consider or report reattachment rate [43]. Another laboratory study forcibly removed partially fed adult female *D. variabilis* ticks from their hosts and infected them with either a growth solution or a solution containing pathogenic material [44]. These ticks were then allowed to reattach and feed to completion [44]. Although *D. variabilis* incomplete feeding studies are limited, studies on other species belonging to the *Dermacentor* genus have been conducted. For example, it has been demonstrated that 47% of male *D. reticulatus* ticks can attach to a new canine host after spending up to 88 hours on the first [45]. In addition, both *D. reticulatus and D. andersoni* ticks have been shown to exhibit intrastadial pathogen transmission, which requires an attached tick to detach and reattach to a new host under natural condition [46,47]. Overall, existing literature, though limited, shows that incomplete feeding has been documented among these, and other closely related species.

Just under half of all mixed blood meal-positive ticks tested positive for at least one pathogen. Despite this, pathogen infection was not a statistically significant risk factor for incomplete feeding. Although this may be due to the overall small sample size, it is important to consider other explanations as well, such as pathogen-induced behavioral changes. Like incomplete feeding, this phenomenon is well studied in other arthropod vectors such as mosquitoes, sandflies, kissing bugs, and fleas [48,49,50,51,52]. While tick studies are more limited, research has shown a tick’s locomotion, questing heights, vertical and horizontal walks, tendency to overcome obstacles, and host-seeking ability are associated with pathogen infection [53]. While there is currently no evidence for this relationship, it is plausible pathogen infection may also influence incomplete feeding among specific tick populations. In addition, it has been suggested that partially fed ticks have a significantly shorter transmission time, allowing them to spread disease more readily [11,54]. Therefore, it is crucial to analyze other tick pathogen types to better understand the mechanisms behind interrupted feeding and pathogenic transmission.

While this study presented several notable preliminary results, it is important to address the potential limitations. First, the tick samples utilized were not representative of the entire state’s tick populations. Only seven months of sampling was conducted between January 2022 and July 2022. During this time, half of all ticks were collected in May, while no ticks were received in April or June, a potential sampling bias. Furthermore, although ticks were collected from each region of SC, only 8 out of 46 total counties were represented in the analysis. Finally, only fully engorged adult female ticks were included. While female ticks take a larger blood meal and become engorged, Ixodidae male ticks are naturally intermittent feeders, suggesting they commonly exhibit incomplete feeding as well [11]. In addition, both larvae and nymphs have demonstrated incomplete feeding behavior [19,20,21,41,42,43]. Therefore, it is likely the results of this study were bound by the small, uniform sample size.

Future studies should utilize a larger sample encompassing all life stages, sex, and engorgement statuses. Another potential limitation arises from the RSID test kits used to detect human blood. Although RSID testing is fast and cost-effective and has previously been used to ascertain human blood in vectors, such as triatomines, the exact sensitivity of the test is not known [55]. Furthermore, more validated vector blood meal testing, such as qPCR and DNA sequencing is available. Future studies should compare these methodologies to find the most reliable test. It is also a possibility that the human blood detected was simply a remanent left over from prior life stages. Therefore, inclusion of all blood feeding life stages is crucial in future studies. Finally, only human blood was directly tested for; however, due to it is preliminary nature, this was beyond the scope of the current study. In an attempt at qPCR blood meal analysis, several primer/probe sequences were screened. These sequences mainly targeted mitochondrial DNA (mtDNA) of humans, dogs, cattle, and deer. These methods and targets have been explored recently in the literature for blood meal identification of arthropods [56]. However, all sequences screened showed cross-reactivity between the previously mentioned animal species using pure species-specific genomic DNA (gDNA) controls purchased from Zyagen, Inc. (San Diego, CA, USA). Although these primer/probe sequences were designed in silico to be species-specific, there is increasing evidence of in vitro cross-reactivity in qPCR methods [25,57,58]. Therefore, further optimization of this protocol or other methods such as next-generation sequencing should be explored. As the tick samples were removed directly from feral dogs, it was assumed they all consumed some amount of canine blood. Future studies should also complete a full comprehensive blood meal analysis, including other common host animals such as cattle, deer, and goats [59]. This will not only greatly expand the scope of the study but will also provide a greater understanding of how many hosts a tick potentially feeds on at each life stage.

## 5. Conclusions

This pilot investigation provided new evidence for incomplete feeding among tick populations in SC. Currently, it is believed ticks feed on a set number of hosts throughout their life [9]. However, this study’s findings suggest otherwise. Seasonality and location are two important risk factors for mixed blood meal-positive ticks. Given that most mixed blood meal-positive ticks were from counties bordering both NC and GA, future surveillance efforts in these areas should be considered. Additionally, it must be considered that pathogen infected, partially fed ticks may be more likely to seek a host, take larger blood meals, and transmit the bacteria at a faster rate [11,54,60,61,62,63,64]. With this, future initiatives to minimize human tick exposure should be implemented, especially in the previously mentioned high risk areas. As global climate change continues to intensify, tick populations will continue to expand their geographic ranges. This range expansion will not only have lasting consequences on tick behavior, but also on human and animal hosts. Incomplete feeding is one adaptation that will allow these ticks to persist in new environments. Therefore, the state of SC must prioritize proactive vector surveillance and control to limit the threat these tick species pose concerning the transmission of tick-borne diseases to human populations. Overall, this investigation has laid the groundwork for future research studies, and surveillance programs to help improve the understanding of this emerging phenomenon.

## Figures and Tables

**Table 1:** Variables associated with mixed blood meal analysis among 225 engorged adult female ticks.

	No. Ticks Human Blood Meal Positive (N = 76)	No. Ticks Human Blood Meal Negative (N = 149)	*p*-Value	OR	95% CI
**Genus**					
*Prostriata*	33 (43.4%)	75 (50.3%)	-	-	-
*Metastriate*	43 (56.6%)	74 (49.7%)	0.327	1.32	0.76 to 2.31
**Species**					
*Amblyomma americanum*	11 (14.5%)	29 (19.5%)	0.356	0.70	0.33 to 1.49
*Dermacentor variabilis*	26 (34.2%)	34 (22.8%)	0.069	1.776	0.96 to 3.23
*Amblyomma maculatum*	0 (0.0%)	5 (3.4%)	-	-	-
*Ixodes scapularis*	25 (32.9%)	64 (43.0%)	0.146	0.65	0.37 to 1.16
*Ixodes kieransi*	8 (10.5%)	11 (7.4%)	0.425	1.48	0.57 to 3.84
*Haemaphyalis longicornis*	6 (7.9%)	6 (4.0%)	0.230	2.04	0.64 to 6.56
**Calendar Month**					
January	22 (28.9%)	44 (29.5%)	0.928	0.78	0.53 to 1.79
February	1 (1.3%)	12 (8.1%)	0.073	0.15	0.02 to 1.19
March	18 (23.7%)	18 (12.1%)	0.027	2.26	1.10 to 4.65
May	33 (43.4%)	74 (49.7%)	0.376	0.15	0.45 to 1.36
July	2 (2.6%)	1 (0.7%)	0.261	0.97	0.36 to 44.83
**Location**					
**Region**					
Lowcountry	25 (32.9%)	68 (45.6%)	0.068	0.58	0.33 to 1.04
PeeDee	18 (23.7%)	22 (14.8%)	0.101	1.79	0.89 to 3.59
Midlands	2 (2.6%)	1 (0.7%)	0.261	3.99	0.36 to 44.83
Upstate	31 (40.8%)	58 (38.9%)	0.787	1.08	0.62 to 1.90
County *					
Aiken	2 (2.6%)	1 (0.7%)	0.261	3.99	0.36 to 44.83
Charleston	25 (32.9%)	68 (45.6%)	0.068	0.58	0.33 to 1.04
Clarendon	0 (0.0%)	4 (2.7%)	-	-	-
Greenville	2 (2.6%)	12 (8.1%)	0.130	0.31	0.07 to 1.41
Greenwood	22 (28.9%)	40 (26.8%)	0.739	1.11	0.60 to 2.05
Horry	18 (23.7%)	18 (12.1.%)	0.027	2.26	1.10 to 4.65
Pickens	6 (7.9%)	6 (4.0%)	0.230	2.04	0.64 to 6.56
York	1 (1.3%)	0 (0.0%)	-	-	-
**Pathogen Testing**					
Overall Total Positives	37 (48.7%)	68 (45.6%)	0.665	1.13	0.65 to 1.97
Panola Mountain *Ehrlichia*	1 (1.3%)	6 (4.0%)	0.263	0.29	0.04 to 2.51
*Ehrlichia ewingii*	1 (1.3%)	4 (2.7%)	0.484	0.45	0.50 to 4.19
*Ehrlichia chaffeensis*	3 (3.9%)	1 (0.7%)	0.127	6.00	0.60 to 59.86
*Rickettsia* species	36 (24.2%)	66 (44.3%)	0.692	1.59	0.16 to 15.87
*Rickettsia parkeri*	17 (11.4%)	31 (20.8%)	0.920	1.04	0.47 to 2.32
*Rickettsia rickettsii*	0 (0.0%)	0 (0.0%)	-	-	-
*Rickettsia amblyommatis*	24 (31.6%)	42 (28.2%)	0.686	1.19	0.52 to 2.72
*Borrelia burgdorferi*	0 (0.0%)	1 (0.7%)	-	-	-
*Anaplasma phagocytophilum*	1 (1.3%)	0 (0.0%)	-	-	-
*Theileria orientalis*	0 (0.0%)	0 (0.0%)	-	-	-

* Category statistically significant overall by multinominal regression.

## Data Availability

The data in this study are available on request from the corresponding author.

## Appendix A

**Table A1:** Primers and Probes for identification of pathogens in ticks by RT-qPCR.

Target Species	Gene	Sequence	Reference
Pathogen			
Panola Mountain *Ehrlichia*	*gltA*	Forward TGTCATTTCCACAGCATTCTCATC	[65]
		Reverse ATTAGCGCAATCATACTTGCAA	
		Probe TGCCTTAGCTGCACATTATTGTGAT	
*Ehrlichia ewingii*	*16S*	Forward GCGGCAAGCCTAACACATG	[65]
		Reverse CCCGTCTGCCACTAACAACTATC	
		Probe TCGAACGAACAATTCCTAAATAGTCTCTGACTATT	
*Ehrlichia chaffeensis*	*16S*	Forward GCGGCAAGCCTAACACATG	[65]
		Reverse CCCGTCTGCCACTAACAATTATT	
		Probe AGTCGAACGGACAATTGCTTATAACCTTTTGGT	
*Rickettsia* species	*23S rRNA*	Forward AGCTTGCTTTTGGATCATTTGG	[66]
		Reverse TTCCTTGCCTTTTCATACATCTAGT	
		Probe CCTGCTTCTATTTGTCTTGCAGTAACACGCCA	
*Rickettsia parkeri*	*ompB*	Forward CAAATGTTGCAGTTCCTCTAAA	[67]
		Reverse AAA ACA AAC CGT TAA AAC TAC CG	
		Probe CGCGAAATTAATACCCTTATGAGCAGCAGTCGCG	
*Rickettsia rickettsii*	*Hypothetical Protein (HP)*	Forward AAATCAACGGAAGAGCAAAAC	[66]
		Reverse CCCTCCACTACCTGCATCAT	
		Probe TCCTCTCCAATCAGCGATTC	
*Rickettsia amblyommatis*	*ompB*	Forward GGTGCTGCGGCTTCTACATTAG	[65]
		Reverse CTGAAACTTGAATAAATCCATTAGTAACAT	
		Probe TCCTCTTACACTTGGACAGAATGCT	
*Borrelia burgdorferi*	*fliD*	Forward TGG TGA CAG AGT GTA TGA TAA TGG AA	[68]
		Reverse ACT CCT CCG GAA GCC ACA A	
		Probe TGC TAA AAT GCT AGG AGA TTG TCT GTC GCC	
*Anaplasma phagocytophilum*	*P44*	Forward ATG GAA GGT AGT GTT GGT TAT GGT ATT	[69]
		Reverse TTG GTC TTG AAG CGC TCG TA	
		Probe TGG TGC CAG GGT TGA GCT TGA GAT TG	
*Theileria orientalis*	*MPSP*	Forward GCA AAC AAG GAT TTG CAC GC	[70]
		Reverse TGT GAG ACT CAA TGC GCC TAG A	
		Probe TCG ACA AGT TCT CAC CAC	
